# P42 Can molecular tests replace conventional methods: rapid detection of bacteraemia and antimicrobial resistance in sepsis: evaluating BioFire BCID2 Panel

**DOI:** 10.1093/jacamr/dlag102.048

**Published:** 2026-06-26

**Authors:** Bharat Chandra Das, Parul Singh, Md Nizam Ahmed, Madhavi Kirti, Aarsi, Kamraan Farooque, Purva Mathur

**Affiliations:** Microbiology Unit, Jai Prakash Narayan Apex Trauma Centre, AIIMS, New Delhi, India; Microbiology Unit, Jai Prakash Narayan Apex Trauma Centre, AIIMS, New Delhi, India; Microbiology Unit, Jai Prakash Narayan Apex Trauma Centre, AIIMS, New Delhi, India; Microbiology Unit, Jai Prakash Narayan Apex Trauma Centre, AIIMS, New Delhi, India; Microbiology Unit, Jai Prakash Narayan Apex Trauma Centre, AIIMS, New Delhi, India; Microbiology Unit, Jai Prakash Narayan Apex Trauma Centre, AIIMS, New Delhi, India; Microbiology Unit, Jai Prakash Narayan Apex Trauma Centre, AIIMS, New Delhi, India

## Abstract

**Background:**

Sepsis is a serious, life-threatening condition which warrants rapid diagnosis and treatment. Molecular tests can save valuable time by their rapid turn-around time and accuracy.

**Objectives:**

To evaluate the diagnostic accuracy of BioFire Blood Culture Identification2 (BCID2) Panel (bioMérieux, France) in detecting bacteraemia and antimicrobial resistance determinants in shortest possible time, as compared to conventional culture-based methods.

**Methods:**

A prospective comparative study was conducted between 2024 and 2025 in Microbiology laboratory, at a level-1 trauma centre in India. 46 blood culture bottles, flagged positive by BACT/ALERT (bioMérieux, France) were analysed. Due to cost considerations, BCID2 testing was performed primarily on samples from critically ill patients. Gram staining was done for every positive bottle, but only those showing Gram-negative bacilli (GNB) or budding yeast cells (BYC) were processed using BCID2 assay. Samples were inoculated on blood agar and MacConkey agar and incubated in ambient air overnight at 37°C. Organisms were identification by VITEK-MS (bioMérieux, France) and antimicrobial susceptibility testing (AST) was done using both disc diffusion and VITEK-2 Compact (bioMérieux, France) were carried out following day. For BCID2 assay samples were processed according to manufacturer’s instructions. Phenotypic culture and AST method was taken as gold standard.

**Results:**

BCID2 assay took about 1 h to provide results from a positive blood culture. Organisms were isolated in 42 out of 46 samples. 2 samples, initially negative by BCID2, showed bacterial growth on culture. *Sphingomonas paucimobilis* and *Corynebacterium striatum* grown on culture next day. Two samples were negative by both methods. BCID2 assay demonstrated a sensitivity of 95.5%, specificity of 100%, positive predictive value of 100%, and negative predictive value of 50%. The agreement between two test by using Cohen’s kappa value showed moderate agreement (k=0.65). Fisher exact test statistic value is 0.0058. The result is significant at *P*<0.05. Eight *Candida* spp. among which *Candida tropicalis* (5/8), *Candida albicans* (2/8) and one *Candida auris* identified by both methods. *Klebsiella pneumoniae* showed 100% concordance, with eight resistant and one susceptible isolate. BCID2 assay failed to detect resistance genes in 3/11 carbapenem-resistant *Acinetobacter baumannii* isolates. Lowest concordance was observed with *Escherichia coli*. Both assays showed similar results in case of only 3 carbapenemase producing Enterobacterales (CRE) isolates. BCID-2 panel detected 6 out of 7 isolates as CRE among which 3 were only ESBL-producing isolates.

**Conclusions:**

Molecular tests are critical for rapid, highly sensitive detection of infectious diseases, genetic disorders, and cancers. At present they are far surpassing traditional culture methods in speed and accuracy. BCID2 panel provides rapid and accurate species identification in bacteraemia patients. Due to multifactorial nature of β-lactam resistance in GNBs, BCID-2 assay should be used in conjunction with conventional phenotypic susceptibility testing for comprehensive AMR detection. We found in our study that BCID2 assay can play an important role in antimicrobial stewardship programme. Most importantly, in case of candidaemia, antifungals were started early. Also, in bacteraemia cases, antibiotic escalation was helpful in decreasing mortality.

